# Comparison of the Metabolic and Flavor Characteristics of the Egg Yolks of BIAN Chicken and Hy-Line Brown Chicken Using LC-MS and GC × GC-TOF MS Techniques

**DOI:** 10.3390/metabo15090609

**Published:** 2025-09-12

**Authors:** Bochi Zhang, Xianyi Song, Kaige Li, Kai Zhang, Rui Zhao, Chunlei Yang, Liying Du

**Affiliations:** College of Animal Science, Shanxi Agricultural University, Taiyuan 030032, China; bochi_zhang@sxau.edu.cn (B.Z.); sxnysxy@163.com (X.S.); 18339464081@163.com (K.L.); zhangkai2255@126.com (K.Z.); zhaorui@sxau.edu.cn (R.Z.); wkyhlycl@126.com (C.Y.)

**Keywords:** BIAN chicken, Hy-Line Brown, egg flavor, metabolomics, flavor compounds

## Abstract

Objectives: This study systematically compared the differences in egg quality between the BIAN chicken, an indigenous breed of Shanxi Province, and the Hy-Line Brown, a commercial breed, through the integration of non-targeted metabolomics and volatile flavoromics methods. Methods: A total of 675 metabolites and 84 volatile flavor compounds were identified in eggs from 300-day-old laying hens using LC-MS and GC × GC-TOF MS techniques. Results: BIAN chicken eggs exhibited notable advantages in flavor quality. The relative odor activity value (ROAV) of 1-octen-3-ol, a key flavor component, was 27.01 in BIAN compared with 13.46 in Hy-Line Brown, contributing to the characteristic mushroom aroma of BIAN eggs. Furthermore, the levels of heptaldehyde, 2-pentylfuran, and styrene in BIAN chicken eggs were significantly elevated, contributing to its characteristic flavor profile. Metabolomic analysis identified 40 breed-specific metabolites in BIAN chicken, with 21 up-regulated and 19 down-regulated. These metabolites were primarily involved in biological processes such as α-linolenic acid metabolism, cholesterol metabolism, and unsaturated fatty acid biosynthesis, highlighting the distinctive lipid metabolism regulation in BIAN chicken. Sensory evaluation based on relative odor activity values (ROAV) demonstrated that BIAN chicken eggs exhibited enhanced sweet, fruity, herbal, and citrus aromas, which correlated with the enriched lipid metabolism pathways. Conclusions: This study elucidates the molecular basis of distinctive egg quality characteristics in local chicken breeds, offering a scientific rationale for the conservation and utilization of indigenous breeds and the documentation of their unique metabolic and sensory properties. Furthermore, it furnishes a theoretical framework for understanding breed-specific flavor development and provides baseline data for future genetic selection and nutritional intervention strategies.

## 1. Introduction

Eggs stand out as a highly nutritious and versatile food source in nature due to their rich composition of bioactive compounds, high-quality proteins, essential fatty acids, vitamins, and minerals. These components collectively enhance the nutritional density and functional characteristics of eggs [1]. Eggs are distinguished by their distinctive composition, featuring a full complement of amino acids. This characteristic confers upon eggs the highest protein digestibility corrected amino acid score, establishing them as the benchmark for evaluating protein quality in the field of nutritional science [2]. In addition to their nutritional value, recent advancements in food science have revealed that eggs contain a diverse array of bioactive compounds such as carotenoids, bioactive peptides, phospholipids, and immunomodulatory proteins. These compounds offer specific health benefits, including cardiovascular protection and cognitive enhancement [3,4,5,6]. Current research has significantly enhanced our comprehension of the functional constituents of eggs, uncovering an intricate network of bioactive compounds that extend beyond fundamental nutrients. For instance, the egg yolk contains notable carotenoids, predominantly lutein and zeaxanthin, which exhibit superior bioavailability compared to plant sources owing to the favorable lipid matrix. The absorption efficiency of these compounds from eggs is 3–4 times greater than that from plant sources. Moreover, these carotenoids have been shown to effectively diminish the likelihood of age-related macular degeneration and improve visual acuity [7]. Notably, egg lipids serve dual roles—providing nutritional benefits while acting as precursors for flavor compounds through oxidation pathways. This nutrition-flavor relationship necessitates integrating analytical methods with sensory evaluation to comprehensively assess egg quality and validate consumer perception of breed-specific differences. Eggs have emerged as a valuable model for investigating the intricate interplay among genetic background, production methods, and food quality attributes. The increasing consumer preference for high-quality egg products with improved nutritional value and superior taste has underscored the importance of exploring breed-specific variations and their associated metabolomic profiles [8].

The impact of breed specificity on egg quality represents a crucial area of research with substantial commercial ramifications. The study found that eggs produced by indigenous Portugal breeds (Branca, Amarela, Pedrês Portuguesa, Preta Lusitânica) had different metabolomic profiles [9]. Comparative analyses indicate that indigenous breeds typically meet or surpass commercial hybrids in specific quality metrics. Furthermore, eggs from native chicken breeds offer nutritional benefits compared to commercial strains, as evidenced by consistent findings across multiple studies demonstrating elevated protein content, favorable fatty acid profiles, and enhanced micronutrient density [10,11]. Central native Moss chickens had higher lipid (9.53%), protein (12.31%) and ash (1.10%) content than commercial Isa Brown strain [12]. The BIAN chicken is a recognized indigenous breed originating from Shanxi Province, China, notable for its moderate body size, distinctive feather pattern, and adaptability to the semi-intensive production system typical of traditional poultry breeding in the region. As a distinct local breed, it has been documented in Chinese poultry genetic resources with characteristic dual-purpose utility for both meat and egg production, yielding an estimated 150–180 eggs annually. Despite its significant genetic diversity and research potential, a comprehensive characterization of this breed is lacking. Recent molecular genetic investigations have highlighted the breed’s distinct genetic and production traits, underscoring its importance for poultry research and conservation initiatives [13]. Preliminary genetic characterization studies using 29 microsatellite markers revealed specific genetic diversity within the breed [14]. Recent research provides specific performance data for Bianchicken, recording that the peak monthly egg production of high-yielding individuals at 228 days of age is 45.33 ± 2.75 eggs, while the peak monthly egg production of low-yielding hens is 26.80 ± 3.08 eggs. The estimated annual egg production is 150–180 eggs per year, positioning this variety as a typical meat and egg dual-purpose category [15]. Given the comprehensive characterization of BIAN chicken varieties, particularly in terms of egg production performance and nutritional composition, systematic investigations are necessary to support conservation efforts and potential commercial exploitation.

The utilization of metabolomics methodologies has transformed the landscape of egg research, facilitating thorough molecular analysis and the identification of biomarkers. Recent seminal investigations have laid down a sturdy methodological groundwork for metabolomics studies on eggs, showcasing a comprehensive non-targeted approach employing UHPLC-MS/MS, which successfully detected 692 metabolites in various egg varieties [16]. A study employed a quasi-targeted metabolomics approach to identify 617 metabolites in chicken, duck, and quail eggs. The study conducted thorough metabolite classification and established the baseline composition profile of eggs [17]. A related study also analyzed 138 egg yolk metabolites and 132 protein metabolites using GC-MS/MS, demonstrating that genetic and environmental factors influence egg composition [18]. Analysis of flavor compounds uncovers intricate flavor compound networks. Recent advancements in volatile compound analysis have transformed our comprehension of egg flavor formation and sensory attributes. Utilizing GC-MS-based volatile metabolomics in conjunction with machine learning algorithms, researchers have pinpointed 11 crucial flavor compounds across various poultry species [19]. Volatile metabolomics was employed for the classification of egg varieties, utilizing SPME-GC-MS to detect 17–18 flavor compounds in shelled eggs, achieving 100% accuracy across three varieties (White Leghorn, Hy-Line Brown, and Jing Fen) in the referenced model; heptanal emerged as a crucial variety-specific marker [20]. This study aimed to delineate breed-specific metabolomic profiles and identify pivotal flavor compounds influencing sensory distinctions in BIAN chicken and Hy-Line Brown eggs. By employing LC-MS for comprehensive non-volatile metabolite profiling and GC × GC-TOF MS for enhanced separation of complex volatile compounds, the research sought to establish analytical methodologies for breed identification and quality evaluation. These findings are crucial for advancing accurate nutritional strategies, guiding genetic selection initiatives, and enhancing egg quality to meet consumer expectations.

## 2. Materials and Methods

### 2.1. Egg Sampling

All animal procedures were approved by the Institutional Animal Care and Use Committee of Shanxi Agricultural University (Approval No. SXAU2023045), and all experimental procedures conducted in accordance with the “Regulations on the Administration of Experimental Animals” approved by the State Council of the People’s Republic of China. BIAN chicken (n = 150) and Hy-Line Brown (n = 150) laying hens were provided by Shanxi Nongkang Xintuo Technology Development Co., Ltd. (Taiyuan, China). All laying hens were 300 days old (approximately 43 weeks, peak laying period) during spring season (March–April) and maintained under identical standardized conditions in environmentally controlled facilities with temperature at 20 ± 2 °C, relative humidity at 60–65%, and a 16L:8D photoperiod. Both breeds were fed the same corn-soybean basal diet formulated, containing 16.5% crude protein, 2750 kcal/kg metabolizable energy, 3.5% calcium, and 0.35% available phosphorus, with the following composition: corn (62.5%), soybean meal (25.2%), limestone (8.5%), dicalcium phosphate (1.5%), salt (0.3%), vitamin-mineral premix (1.0%), and soybean oil (1.0%), with feed and water provided ad libitum. Eggs were collected within 2 h of laying during the morning collection period (08:00–10:00) to minimize metabolite variations due to storage time, with a total of 180 eggs (90 from each breed) collected from randomly selected healthy hens with similar body weights. Only clean, intact eggs with normal shell quality were selected and immediately transported to the laboratory in a temperature-controlled container and stored at 4 °C for no more than 24 h before processing. Twelve eggs were randomly selected (equal numbers per breed) from each group, brought to room temperature (20 °C) for 30 min before processing, and after careful separation of albumen and yolk using standard techniques, yolk samples were homogenized, aliquoted into 2 mL cryovials, flash-frozen in liquid nitrogen, and stored at −80 °C until analysis within one month, with 6 eggs used for LC-MS metabolomic analysis and 6 eggs used for GC × GC-TOF MS analysis.

### 2.2. Chemical Reagents and Instruments

The main reagents used were methanol (HPLC grade), chloroform (analytical grade), acetonitrile (LC-MS grade), and 2-chloro-L-phenylalanine (4 ppm, internal standard). Additional reagents included ethanol (analytical grade), deuterated n-hexanol-d13 (used as an internal standard for GC), n-alkane (for retention index calibration), and n-hexane (GC grade). All the reagents were obtained from Shanghai Baiqu Biomedical Technology Co., Ltd. (Shanghai, China). The LC analysis was performed on a Vanquish UHPLC System (Thermo Fisher Scientific, Waltham, MA, USA), Mass spectroscopic detection of metabolites was performed on Q Exactive Focus (Thermo Fisher Scientific, Waltham, MA, USA); analyses were carried out using a LECO Pegasus^®^ 4D instrument (LECO, St. Joseph, MI, USA) consisting of an Agilent 8890A GC (Agilent Technologies, Palo Alto, CA, USA).

### 2.3. Two-Dimensional Gas Chromatography-Time of Flight Mass Spectrometry (GC × GC-TOF MS) Analysis

Sample preparation: Take 3.0 g of egg yolk sample into a 20 mL headspace injection vial, and add 10 μL of internal standard solution for quantitative calibration. Samples were incubated at 80 °C for 10 min to allow sufficient release of volatile compounds into the headspace. SPME extraction heads (DVB/CAR/PDMS coating: Divinylbenzene/Carboxen/Polydimethylsiloxane (obtained from Shanghai Baiqu Biomedical Technology Co., Ltd. (Shanghai, China)), 50/30 μm film thickness) are aged at 270 °C for 10 min prior to use to remove possible contaminants. The aged SPME extraction head is inserted into the headspace of the sample bottle and adsorbed at 80 °C for 25 min to ensure adequate concentration of volatile compounds. After adsorption, transfer the SPME extraction head to the gas chromatography injection port and thermally desorb at 250 °C for 5 min to completely release the adsorbed compounds into the chromatographic system. After each sample injection, the SPME extraction head is aged at 270 °C for 10 min in preparation for the next analysis. To calculate the retention index, 10 μL of n-alkane standard (C7–C30) was placed in a 20-mL headspace injection vial and incubated, extracted, and injected for analysis under the same conditions. This n-alkane standard was analyzed once per analytical batch for retention index calculation. LECO Pegasus BT 4D full 2D gas chromatography-time of flight mass spectrometer was used. The system consisted of Agilent 8890A gas chromatograph, two-stage injection modulator, split/splitless injection module and high resolution TOF mass spectrometer detector. Orthogonal double column system was used for chromatographic separation: DB-Heavy Wax polar column (30 m × 250 μm × 0.5 μm) was used for one-dimensional chromatography, and separation was mainly carried out according to the polarity of compounds; Rxi-5Sil MS non-polar column (2 m × 150 μm × 0.15 μm) was used for two-dimensional chromatography, and secondary separation was mainly carried out according to the boiling point of compounds. This orthogonal separation mode can significantly improve the resolution and peak capacity of complex samples. Chromatographic conditions were set as follows: high purity helium was used as carrier gas at a constant flow rate of 1.0 mL/min. One-dimensional column temperature program: initial temperature 50 °C, hold for 2 min, 5 °C/min to 230 °C, hold for 5 min. The 2D column temperature is always 5 °C higher than the 1D column temperature to prevent condensation of compounds between columns. The modulator temperature was maintained at 15 °C above the 2D column and the modulation period was set to 6.0 s to ensure complete cleavage of the 1D effluent and rapid separation in 2D. The injection port temperature was set to 250 °C and splitless injection mode was used. LECO Pegasus BT 4D time-of-flight mass spectrometer was used for mass spectrometry detection. The operating parameters were set as follows: transmission line temperature 250 °C, ion source temperature 250 °C, electron bombardment ionization energy 70 eV, detector voltage 1960 V. Mass spectrum scanning range *m*/*z* 35–550, collection rate 200 spectra/s high-speed acquisition mode, to ensure accurate detection of 2D chromatography fast elution peak [20].

### 2.4. LC-MS Metabolomic Analysis

Metabolite extraction of egg yolk samples was performed in methanol-chloroform-water system (75% 9:1 methanol: chloroform, 25% H_2_O). After grinding at 50 Hz in tissue grinder, sonication at room temperature for 30 min and ice bath for 30 min, the supernatant was collected and concentrated for drying at 12,000 rpm and 4 °C. Finally, 200 μL of 50% acetonitrile solution containing internal standard (4 ppm 2-chloro-L-phenylalanine) was reconstituted and filtered for LC-MS analysis. Quality control (QC) samples were prepared by pooling equal aliquots from all egg yolk samples and were injected every 10 samples to monitor system stability. Blank samples (extraction solvent only) were analyzed at the beginning and end of each batch to assess background contamination. Compound identification was based on the following criteria: (1) mass accuracy < 5 ppm for precursor ions, (2) retention time matching with authentic standards when available, (3) MS/MS spectral matching with reference databases, and (4) isotope pattern matching. Metabolites meeting at least three of these criteria were considered confidently identified. Chromatographic separation was performed on a Thermo Vanquish Ultra Performance Liquid Chromatography system (Thermo Fisher Scientific, Waltham, MA, USA) equipped with an ACQUITY UPLC^®^ HSS T3 reverse-phase column (2.1 × 100 mm, 1.8 μm; Waters, Milford, MA, USA) at a flow rate of 0.3 mL/min, column temperature of 40 °C, and injection volume of 2 μL. For positive ion mode, 0.1% formic acid water (A2) and 0.1% formic acid acetonitrile (B2) were used as mobile phases, and for negative ion mode, 5 mM ammonium formate water (A3) and acetonitrile (B3) were used as mobile phases. For both modes, an 8-min gradient elution procedure was used, and the organic phase ratio was linearly increased from 10% to 98% (1–5 min). After 1.5 min, the initial conditions were quickly returned to equilibrium. Mass spectrometry was performed using a Thermo Q Exactive Focus high-resolution mass spectrometer (Thermo Fisher Scientific, Waltham, MA, USA) equipped with an electrospray ionization source (ESI) with independent acquisition in positive and negative ion mode. The ion source parameters were set as follows: positive ion spray voltage 3.50 kV, negative ion spray voltage −2.50 kV, sheath gas flow rate 40 arb, auxiliary gas flow rate 10 arb, and capillary temperature 325 °C. Data acquisition was performed in Full MS/dd-MS^2^ mode, with a full scan resolution of 70,000 for primary mass spectrometry and a scan range of *m*/*z* 100–1000; data dependent acquisition (DDA) was used for secondary mass spectrometry, with the parent ions in the top 3 positions of intensity selected for high energy collision dissociation (HCD) fragmentation, collision energy 30 eV, secondary resolution 17,500, and dynamic exclusion enabled to improve metabolite coverage [18].

### 2.5. Statistic Analysis

Six biological replicates (n = 6) were analyzed for each group in both LC-MS metabolomics and GC × GC-TOF MS analyses. The t-test and one-way analysis of variance (ANOVA) were used to assess the significance of differences between groups. Results are expressed as mean ± standard error (SEM). To control the *p* value for multiple comparisons in metabolites, the false discovery rate was determined using the approach of Benjamini and Hochberg. Raw data from GC × GC-TOF MS was processed using ChromaTOF software (version 4.71) for peak alignment (RT tolerance: ±0.5 s for 1D, ±0.05 s for 2D) and compound identification against the NIST2020 mass spectral library with minimum similarity score ≥800 and retention indices calculated using n-alkane standards (C7–C30). For LC-MS data, Compound Discoverer 3.3 was used for peak alignment (mass tolerance: 5 ppm, RT tolerance: 0.2 min) and metabolite annotation was performed using HMDB, KEGG, LipidMaps, and mzCloud databases, with identification confidence levels assigned according to Metabolomics Standards Initiative guidelines. Images were created using Rstudio (version 2023.12.1) and GraphPad Prism (version 7.0). Loading algorithms can identify the contribution of each sensor to each experimental sample discrimination. Principal component analysis (PCA) and Kyoto Encyclopedia of Genes and Genomes pathway analysis were performed using MetaboAnalyst (www.metaboanalyst.ca (accessed on 8 June 2024)) 5.0. Principal component analysis (PCA) and partial least squares discriminant analysis (PLS-DA) were performed at metaX (version 1.4.16). Metabolites with different importance, such as predicted (VIP) > 1,FDR-adjusted *p*-value < 0.05, fold change (FC) ≥ 2, or FC ≤ 0.5, were considered differential metabolites.

## 3. Results

### 3.1. Analysis of Volatile Flavor Compounds in Egg Yolk

**Figure 1:**
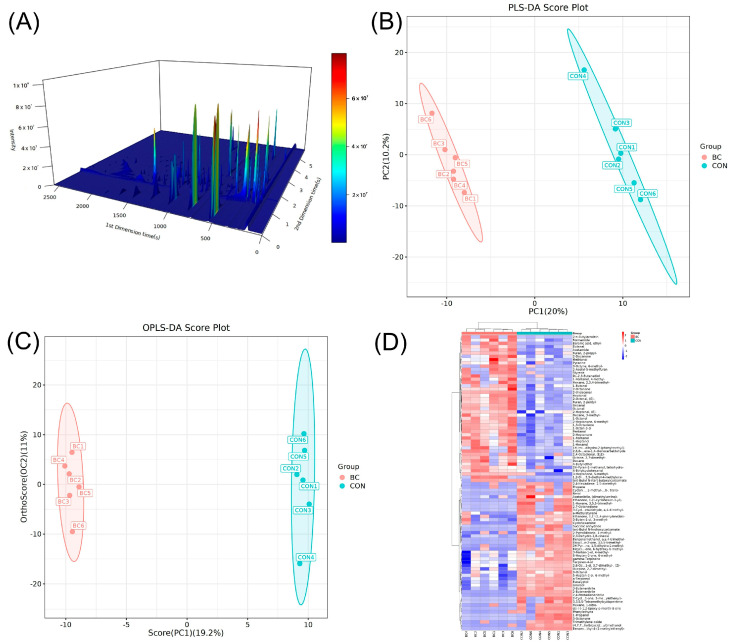
GC × GC-TOF analysis of egg yolks from BIAN chicken (BC) and Hy-Line Brown chicken (CON). (**A**) Chromatographic 3D plot of egg yolk samples identified by GC × GC-TOF MS. (**B**) PLS-DA score chart. (**C**) OPLS-DA score chart. (**D**) Heat maps of differentially flavored compounds. BC: BIAN chicken; CON: Hy-Line Brown chicken.

Volatile flavor components in egg yolk samples (n = 6) were analyzed using GC × GC-TOF MS. The chromatogram obtained from GC × GC-TOF MS is presented in Figure 1A. Multivariate statistical analysis revealed significant differentiation in volatile flavor components among eggs sourced from distinct origins, as illustrated in Figure 1B,C. PLS-DA, incorporating breed category data, exhibited complete segregation of the two breeds along the first principal component (PC1). Notably, the BIAN chicken samples cluster separately from the Hy-Line Brown samples, appearing in the lower-left quadrant of the scores plot (i.e., with relatively lower PC1 and PC2 scores). This indicates that BIAN samples are biochemically distinct in those principal components from the Hy-Line Brown samples. The OPLS-DA score plot further accentuated the inter-group discrimination, displaying a pronounced bipolar distribution along the horizontal axis. Within-group sample points exhibited tight clustering, while the inter-group boundary remained distinctly demarcated, underscoring systematic disparities in volatile components between breeds and robust biological reproducibility. The raw data obtained from the instrument underwent flavor compound annotation analysis using the Chroma TOF library search software in conjunction with the NIST2020 database. This analysis facilitated the detailed identification of flavor compounds present in the egg yolks of BIAN chicken and Hy-Line Brown. A total of 84 aroma molecules with corresponding CAS numbers were identified in the study. These compounds were categorized based on their chemical structures, revealing the following distribution: 14 alcohols, 13 ketones, 11 aldehydes, 11 hydrocarbons, 10 organic heterocyclic compounds, 8 lipids and lipid-like molecules, 6 benzene-ring compounds, and 4 nitrogen-containing compounds, among others. Statistical tests revealed that compared with the Hy-Line Brown breed, the concentrations of 42 aroma molecules in the eggs of the BIAN chicken breed increased, while those of another 42 molecules decreased. The visualization results of the heatmap indicated that BIAN chicken egg products may possess unique flavor components (Figure 1D) distinct from those of commercial laying hens. The heatmap revealed a remarkable breed-specific expression pattern of differential flavor compounds between BIAN and Hy-Line Brown eggs. The clear separation in the dendrogram confirmed excellent reproducibility within the breeds and the fundamental metabolic differences between them.

### 3.2. Screening of Differential Flavor Compounds in the Egg Yolks of BIAN Chicken and Hy-Line Browns

Following noise filtering, we acquired the identification details of flavor compounds. The identification results revealed that the predominant flavor compounds in the egg yolks of each sample were primarily alcohols, aldehydes, organic acids, and their derivatives. Through relative odor activity values (ROAVs) analysis, we systematically assessed the contributions and odor characteristics of key flavor compounds in BIAN chicken and Hy-Line Brown egg yolks. Table 1 displays the differential flavor compounds identified, with BIAN chicken eggs exhibiting elevated levels of Styrene (CAS: 100-42-5), (E)-2-Heptenal (CAS: 18829-55-5), (E)-2-Octenal (CAS: 2548-87-0), Heptanal (CAS: 111-71-7), 1-Octen-3-ol (CAS: 3391-86-4), 2-Octanone (CAS: 111-13-7), 2-pentyl-Furan (CAS: 3777-69-3), Decanal (CAS: 112-31-2), Pentanal (CAS: 110-62-3), 1-Butanol (CAS: 71-36-3), and other compounds. The primary aroma-contributing compound identified in BIAN chicken egg yolks, determined through ROAV analysis, was 1-octen-3-ol, which significantly influenced the distinctive aroma profile, with a notably high ROAV value of 27.01 compared to Hy-Line Brown eggs (ROAV 13.46), imparting a characteristic mushroom aroma. This compound likely plays a crucial role in defining the unique flavor of BIAN chicken eggs. Additionally, other compounds demonstrated substantial flavor contributions in BIAN chicken egg yolks, including Heptanal (ROAV 5.31, Hy-Line Brown 2.53) with citrus and fatty notes, 2-pentyl-Furan (ROAV 5.05, Hy-Line Brown 1.57) evoking green bean and vegetable aromas, and 1-Butanol (ROAV 4.76, Hy-Line Brown 3.54) offering sweet, malt, and alcoholic flavors. Furthermore, compounds like (E)-2-Octenal (ROAV 1.37 vs. 0.41 in Hy-Line Brown) and Decanal (ROAV 2.45 vs. 0.77 in Hy-Line Brown) also exhibited notable flavor contributions in BIAN chicken egg yolks. A thorough analysis reveals that the superior flavor of BIAN chicken eggs is primarily attributed to 1-Octen-3-ol. This compound, known for its mushroom-like aroma, along with the collaborative influence of other compounds featuring fatty, green, and sweet notes, collectively shapes the distinctive flavor profile of BIAN chicken eggs.

### 3.3. Sensory Flavor Analysis of Egg Yolks from BIAN Chicken and Hy-Line Brown Chicken

**Figure 2:**
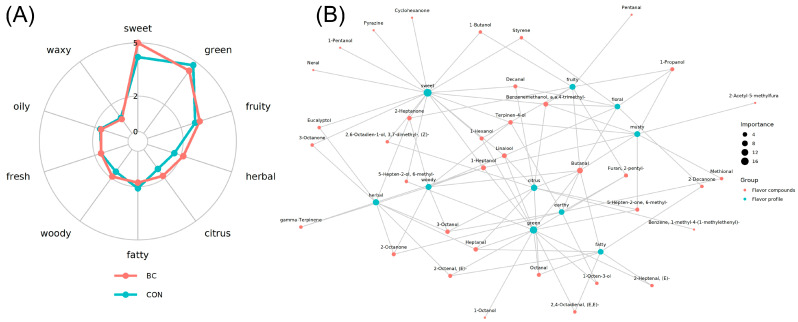
(**A**) Radar chart for sensory flavor characteristic analysis. (**B**) Correlation network diagram between sensory and flavor compounds.

The radar chart depicting sensory flavor analysis (Figure 2A) illustrated the distinctions and similarities between BIAN and Hy-Line Brown eggs across ten flavor attributes. Figure 2A demonstrated discernible differences in the overall flavor profiles of the two breeds. Particularly noteworthy was the pronounced divergence in sweetness, with BIAN exhibiting significantly higher intensity than Hy-Line Brown, suggesting a potentially richer presence of sweet volatile compounds in BIAN. Additionally, BIAN excels in fruity, herbal, citrus, and woody attributes. Conversely, Hy-Line Brown registers notably higher intensity in green and fatty characteristics compared to BIAN. The graph software was utilized to create a network diagram illustrating the relationships between flavor compounds and sensory attributes. In Figure 2B, red circles denote flavor compounds, with larger circles indicating a higher number of associated sensory characteristics. Green circles represent sensory attributes, and their size correlates with the diversity of flavor compounds linked to each specific attribute. This analysis highlights the interplay between volatile organic compounds (VOCs) and flavor, suggesting that the characteristic volatile compound profile of BIAN eggs is attributed to the relatively elevated expression of VOCs. Analysis using GC × GC-TOF MS to differentiate between the two egg types revealed the unique flavor profile of BIAN chicken eggs.

### 3.4. LC-MS Metabolomic Analysis of Egg Yolks from BIAN Chicken and Hy-Line Brown Chicken

**Figure 3:**
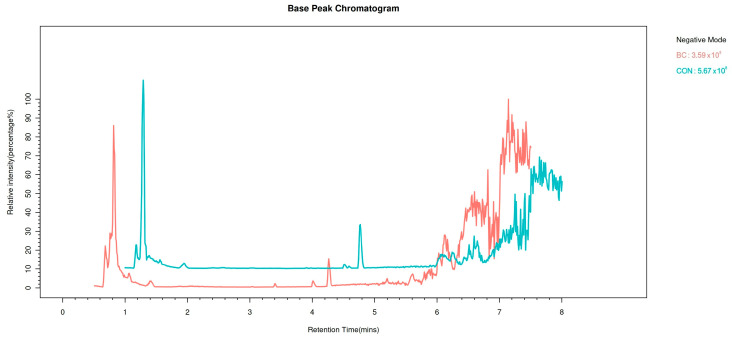
Total ion chromatogram-mass spectra of egg yolks from the corrected BIAN chicken and Hy-Line Brown chicken.

Differences in specific volatile flavors among egg products from distinct chicken breeds prompted an investigation into the metabolomic profiles of BIAN chicken and Hy-Line Brown egg yolks. An untargeted metabolomics analysis was performed using liquid chromatography-mass spectrometry to characterize their small molecule compositions. Figure 3 displays the total ion chromatogram in positive ion scanning mode. A total of 675 metabolites were identified across all egg samples using LC-MS analysis. To identify metabolites with significant differences between breeds, we applied a two-step screening approach. First, using initial statistical criteria (*p* < 0.05 and VIP > 1), 113 metabolites showed differential expression between BIAN chicken and Hy-Line Brown eggs. Of these 113 differential metabolites, 45 could not be annotated due to database limitations, while 68 were successfully identified, including lipids, benzene derivatives, and organic heterocyclic compounds. In the egg yolks of BIAN chicken and Hy-Line Brown, 53 metabolites were identified, comprising 27 lipids and lipid-like molecules, 6 organic oxygen compounds, 7 benzenoids, 5 organoheterocyclic compounds, and other metabolites.

### 3.5. Multivariate Statistical Analysis of Differential Metabolites in Egg Yolks

**Figure 4:**
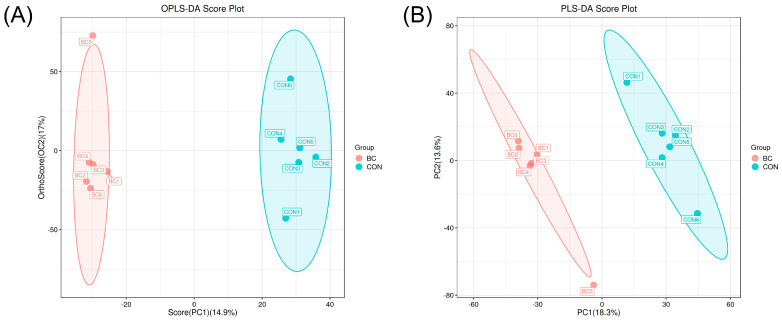
Principal component analysis of the flavor substance contents in the egg yolks of BIAN chicken and Hy-Line Brown chicken. (**A**) PLS-DA score chart, (**B**) OPLS-DA score chart.

Initially, principal component analysis (PCA) was employed to investigate the data structure, detect sample distribution patterns, and identify potential outliers. Due to the limited discriminative capacity of unsupervised dimensionality reduction techniques, partial least squares discriminant analysis (PLS-DA) was subsequently applied to enhance classification accuracy. The PLS-DA model, utilizing chromatographic peak areas, effectively discerned variety-specific metabolic markers. By employing PLS-DA on chromatographic areas, metabolites distinguishing between the BIAN chicken and Hy-Line Brown egg groups were identified. Model performance assessment results (Figure 4) are as follows: R2Y = 0.992 and Q2 = 0.734 for the PLS-DA model, and R2Y = 0.991 and Q2 = 0.545 for the orthogonal partial least squares discriminant analysis (OPLS-DA) model. Classification predictions were made by establishing a quantitative relationship between metabolite levels and variety classifications. The models’ robustness was confirmed through permutation tests, mitigating concerns of overfitting. Both discriminant models exhibited strong predictive capabilities, underscoring distinct metabolic phenotypic variances between the BIAN chicken and Hy-Line Brown egg groups, thus highlighting differences in their metabolite profiles.

### 3.6. Identification of Differential Metabolites in the Egg Yolks of BIAN Chicken and Hy-Line Brown Chicken

**Figure 5:**
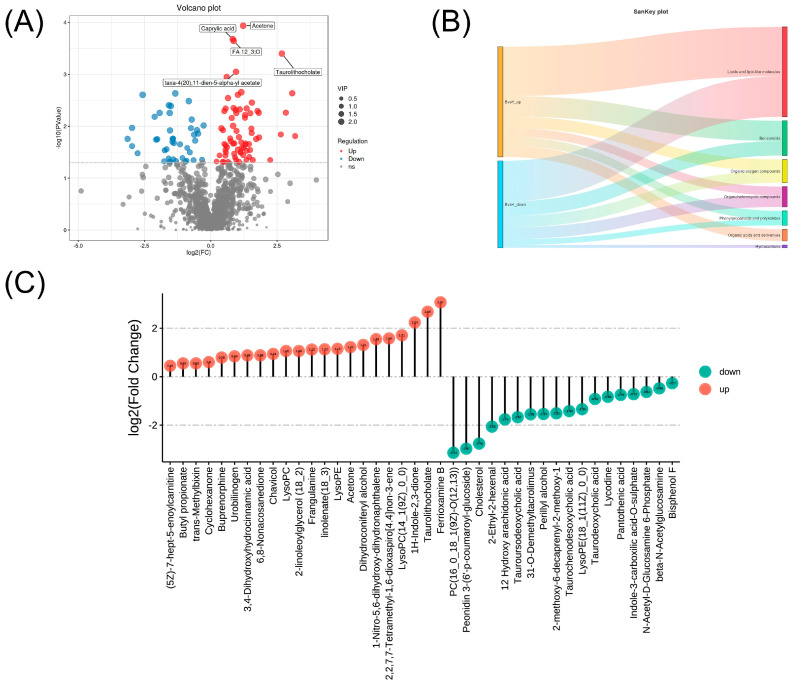
(**A**) Volcano plot showing the differences among different metabolites. (**B**) Sankey diagram for the classification of differential metabolites. (**C**) Lollipop chart presenting the top 20 differential metabolites between BIAN chicken and Hy-Line Brown chicken. FC: Fold Change; VIP: Variable Importance in Projection.

Metabolomic analysis systematically compared egg yolk metabolite profiles from BIAN and Hy-Line Brown chicken. Using stringent criteria (Variable Importance in Projection (VIP) > 1, False Discovery Rate (FDR)-adjusted *p* < 0.05, log2 (Fold Change) ≥ 1 or ≤−1), 40 breed-specific metabolites were identified (Figure 5). The volcano plot (Figure 5A) showed clear separation, with 21 metabolites upregulated (red dots) and 19 downregulated (blue dots) in BIAN egg yolks. The *x*-axis represents log2 (Fold Change), and the *y*-axis represents −log10 (FDR-adjusted *p*-value), with dashed lines indicating thresholds. Most differentially expressed metabolites exhibited fold changes between 2 and 8, underscoring substantial metabolic distinctions between the breeds. The mulberry plot (Figure 5B) depicted the classification and expression patterns of these metabolites across the two breeds. The main differences were concentrated in the following categories: Lipid-related molecules were dominant (17 were highly expressed and 14 were lowly expressed in BIAN chicken), accounting for 77.5% of the total differential metabolites; all organic acid derivatives were highly expressed in BIAN chicken (4 in total), suggesting that BIAN chicken may have more active organic acid metabolism; the distribution of metabolites in other categories was relatively uniform. Based on the 2-fold change threshold, the top 20 metabolites significantly enriched in the egg samples of BIAN chicken were: Ferrioxamine B, Taurolithocholate, 1H-Indole-2,3-dione, Dihydroconiferyl alcohol, Acetone, linolenate (18_3), Frangulanine, 2-linoleoylglycer, Chavicol, 3,4-Dihydroxyhydrocinnamic acid, 6,8-Nonacosanedione, Urobilinogen, Buprenorphine, Cyclohexanone, Butyl propionate, trans-Methylbixin, etc. These metabolites covered multiple biological processes such as fatty acid metabolism, amino acid metabolism, and secondary metabolism. Notably, multiple unsaturated fatty acids and their derivatives (such as linolenate and 2-linoleoylglycer) were significantly enriched in BIAN chicken, suggesting that the egg yolk of BIAN chicken may have higher nutritional value. From a human health perspective, the elevated α-linolenic acid (18:3) may provide cardiovascular benefits and anti-inflammatory effects, while 2-linoleoylglycerol could contribute to improved lipid metabolism and potential neuroprotective properties. The higher levels of phenolic compounds like 3,4-Dihydroxyhydrocinnamic acid may offer enhanced antioxidant protection against oxidative stress-related diseases.

### 3.7. Pathway Analysis of Differential Metabolites

**Figure 6:**
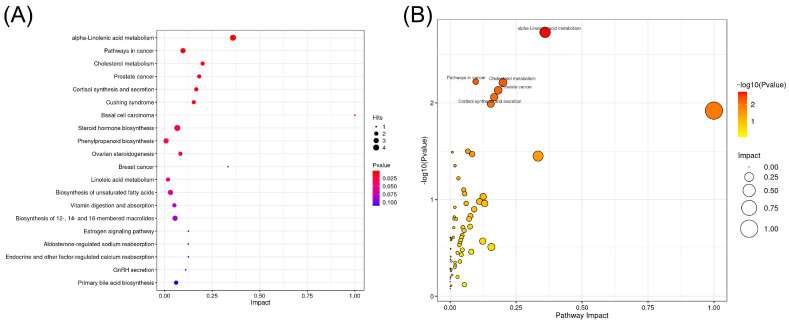
Functional analysis of differentially regulated metabolites. (**A**) Metabolite set enrichment analysis, (**B**) Kyoto Encyclopedia of Genes and Genomes pathway analysis.

Metabolite enrichment and Kyoto Encyclopedia of Genes and Genomes (KEGG) pathway analyses were conducted on the differentially expressed metabolites, with results presented in Figure 6. The metabolite enrichment analysis (Figure 6A) identified several significantly enriched metabolic pathways (*p* < 0.05): Alpha-Linolenic acid metabolism emerged as the most significantly enriched pathway, indicating notable variations in essential fatty acid metabolism. Cholesterol metabolism highlighted distinctions in lipid synthesis and transport between the two varieties. Phenylpropanoid biosynthesis was associated with the synthesis of diverse bioactive compounds. Furthermore, Linoleic acid metabolism and Biosynthesis of unsaturated fatty acids underscored the pivotal role of lipid metabolism. The KEGG pathway analysis, depicted in the bubble chart of Figure 6B, illustrated the enrichment degree and significance of the pathways. In this visualization, the horizontal axis (Pathway Impact) ranged from 0 to 1.0, with values closer to 1.0 indicating greater pathway influence. The vertical axis showed −log10 (*p*-value), where higher values indicated greater statistical significance (*p* < 0.05 corresponded to values above 1.3). Bubble sizes were proportional to the number of differentially expressed metabolites in each pathway, with larger bubbles containing more metabolites. Bubble colors transitioned from yellow to red, with deeper red indicating higher impact scores. The three most prominent bubbles in the upper right quadrant represented α-linolenic acid metabolism (Pathway Impact: 0.65, containing 8 metabolites), cholesterol metabolism (Pathway Impact: 0.55, containing 6 metabolites), and biosynthesis of unsaturated fatty acids (Pathway Impact: 0.45, containing 7 metabolites). These pathways appeared as large, red bubbles positioned in the high-impact, high-significance region of the chart. The enrichment of these core pathways indicates that there are systematic differences between BIAN chicken and Hy-Line Browns in key physiological processes such as lipid metabolism regulation and hormone synthesis. Comprehensive analysis suggests that the egg yolks of BIAN chicken exhibit unique metabolic characteristics in lipid metabolism, especially in unsaturated fatty acid metabolism, which may serve as an important material basis for their special nutritional qualities and flavor characteristics.

## 4. Discussion

In this study, 84 volatile compounds were identified using GC × GC-TOF MS analysis. Of these, 42 compounds exhibited higher concentrations in BIAN chicken eggs, while 42 compounds showed lower concentrations in Hy-Line Brown chicken eggs, indicating notable breed-specific variations in egg flavor chemistry. These breed-specific variations align with comparative studies between Leghorn and other heritage breeds, where indigenous chickens consistently demonstrate unique volatile compound profiles and superior sensory characteristics compared to commercial strains, reinforcing the importance of preserving local genetic resources for high-quality egg production [9,18]. The distinct flavor profiles observed between BIAN chicken eggs and Hy-Line Brown chicken eggs are primarily attributed to specific volatile organic compounds (VOCs) that influence sensory perception through their unique chemical properties and formation pathways. Aldehydes, predominantly originating from lipid oxidation processes, constitute the primary category of volatile compounds in eggs [21]. Notably, Nonanal, a product of linoleic acid and oleic acid, is notably elevated in DHA-enriched egg yolks. The integrated analysis of lipidomics and volatile compounds elucidates the interplay between lipids and flavor in DHA-enriched egg yolks, indicating that optimizing the fatty acid composition in traditional breeds enhances the production of aldehyde compounds [22]. In our study, both BIAN chicken and Hy-Line Brown laying hens were fed identical corn-soybean diets under similar environmental conditions, suggesting that the observed differences in n-3 PUFA content and the enrichment of α-linolenic acid metabolism pathway (*p* < 0.05) are likely intrinsic to the BIAN breed rather than diet-derived. This breed-specific metabolic advantage is further supported by the significant upregulation of unsaturated fatty acid biosynthesis and linoleic acid metabolism pathways in BIAN chicken, indicating that indigenous breeds like BIAN chicken may possess enhanced genetic capacity for n-3 PUFA synthesis and deposition in eggs. Valeraldehyde produces a pleasant almond scent in low concentrations, underscoring the significance of concentration-dependent sensory perception [23]. Decanal maintains its floral and citrus characteristics throughout the heating process, enriching the overall taste of the egg yolk. Heptanal contributes green, citrus, fatty, and nutty aromas, accompanied by fruity and sweet elements. Heptanal serves as a natural flavor enhancer, with its levels notably higher in traditional breeds, especially in response to increased DHA content, whereas commercial breeds generally lack this compound [22]. The combined effects of these aldehydes result in a sophisticated flavor profile that cannot be replicated by any single compound, as evidenced in prior research on the interactions of volatile compounds [24].

Alcohols, ketones, furans, and aromatic compounds found in BIAN chicken eggs offer diverse flavor profiles due to distinct formation pathways and sensory attributes. A significant differentiating factor is 1-Octen-3-ol, known for its mushroom-like aroma, contributing earthy, fungal, green, greasy, and vegetal notes. This compound enhances umami and broth-like flavors, playing a crucial role in defining the high-quality egg taste, particularly between different breeds [25]. Interestingly, 1-octen-3-ol levels vary considerably across poultry species: duck eggs typically contain lower concentrations (ROAV 8–10) compared to chicken eggs, while quail eggs show minimal levels (ROAV < 5), contributing to their distinct milder flavor profiles. The notably high ROAV of 27.01 in BIAN chicken eggs exceeds not only commercial chicken breeds but also surpasses levels reported in other traditional chicken breeds and poultry species, establishing it as a unique flavor marker. When cooked at the ideal temperature, traditional eggs exhibit more favorable volatile flavor characteristics, such as 2-pentylfuran, which adds buttery, floral, fruity, and green bean notes to the eggs. The formation of this furan compound is primarily attributed to lipid oxidation from the breakdown of linoleic acid hydroperoxides. Traditional breeds demonstrate higher efficiency in converting substrates to end products [26]. Analysis from sensory evaluations reveals that BIAN chicken eggs possess stronger sweet, fruity, herbaceous, citrus, and woody aromas due to elevated concentrations of specific volatile compounds identified through GC × GC-TOF MS analysis. Consumer preference studies confirm the significance of these volatile compounds through objective assessments. Research has demonstrated that Hy-Line Brown commercial eggs exhibit lower scores in milk flavor, moisture, and compactness compared to Chinese native breeds [27]. These findings underscore the importance of preserving and utilizing indigenous chicken breeds to safeguard genetic diversity and distinctive flavor profiles in egg production. Future investigations should prioritize elucidating the molecular mechanisms underlying breed-associated differences in yolk volatile profiles, with particular emphasis on identifying genetic and regulatory determinants that impact lipid metabolism and flavor compound biosynthesis.

Understanding the metabolomic composition of eggs has emerged as a significant frontier in poultry science, food technology, and human nutrition. Recent advancements in analytical chemistry, particularly liquid chromatograph-mass spectrometry (LC-MS), have unveiled the complex biochemical landscape of eggs, which encompasses hundreds of metabolites influencing flavor, nutrition, and health benefits. Among the target metabolites, linolenic acid shows strong correlation with egg flavor formation, potentially acting through established lipid oxidation pathways. This essential ω-3 fatty acid may serve as a precursor for volatile aldehyde formation during heat treatment through enzymatic lipoxygenase activity and subsequent hydroperoxide decomposition, though direct causality remains to be experimentally validated. While our data demonstrate significant associations between linolenic acid content and specific flavor compounds (hexanal, nonanal), the mechanistic link requires further validation through controlled enzymatic studies. Previous research has suggested linolenic acid as an important pathway for flavor development [28,29], supporting our correlative findings. From the perspective of flavor chemistry, linoleic acid acts as a key precursor for important volatile compounds through the lipid oxidation process. During the autoxidation process in cooking or processing, linoleic acid forms aldehydes, including 2-nonenal, 2,4-decadienal, hexanal, octanal, and nonanal, which contribute to the pleasant flavor of cooked egg products [30,31]. 2-Linoleoylglycerol, a novel bioactive compound of emerging importance, plays a role in egg nutrition. As a monoacylglycerol of linoleic acid, it acts as a partial agonist of the cannabinoid CB1 receptor, potentially regulating metabolic pathways and providing neuroprotective effects [32,33].

The detection of 3,4-dihydroxybenzoic acid in egg yolk samples is a significant discovery, which is related to the antioxidant capacity and potential health benefits of eggs from different chicken breeds. In food systems, phenolic compounds such as 3,4-dihydroxybenzoic acid offer multiple benefits, including free radical scavenging activity, metal chelating ability, and antibacterial properties [34]. The detection of trans-Methylbixin in our LC-MS analysis represents an interesting finding regarding carotenoid compounds in the egg yolk system. This methyl derivative of curcumin exhibits higher bioavailability compared to the parent compound while maintaining anti-inflammatory, antioxidant, and antibacterial activities [35,36]. In food applications, trans-methylcurcumin serves both as a natural colorant, providing a yellow pigment, and as a bioactive compound with documented health benefits [37]. The source and biological significance of trans-Methylbixin in BIAN chicken eggs warrant further investigation. Given that both breeds were reared under identical conditions and fed the same corn–soybean diet, the between-breed differences in yolk phenolic profiles are more plausibly attributable to breed-related physiological regulation (absorption, conjugation, transport, and deposition) rather than dietary variation. Acetone was detected as a naturally occurring metabolite significantly enriched in BIAN chicken egg yolks. As an endogenous ketone body, acetone can be produced through fatty acid β-oxidation and ketogenesis pathways in biological systems. This compound contributes a fruity and ethereal note to the overall flavor profile. The differential expression of acetone between BIAN chicken and Hy-Line Brown eggs suggests possible variations in lipid metabolism efficiency or ketone body production between breeds, potentially reflecting differences in fatty acid oxidation capacity [38]. The coordinated enrichment of multiple interconnected metabolic pathways in our comparative analysis indicates that breed-specific differences in egg yolk composition stem from comprehensive metabolic adaptations rather than isolated pathway modifications. The simultaneous enrichment of α-linolenic acid metabolism, cholesterol metabolism, cortisol synthesis, phenylpropanoid biosynthesis, linoleic acid metabolism, and unsaturated fatty acid biosynthesis indicates a systematic metabolic reorganization, which reflects fundamental differences between indigenous and commercial chicken breeds in responding to the complex physiological challenges of egg formation [39,40]. Our study’s integrated metabolic pathways enhance understanding of egg quality and nutrition. We elucidated BIAN chicken eggs’ superior flavor and nutritional value through metabolomic and flavoromic analyses, supporting conservation and utilization of local breeds for high-quality egg production. These breed-specific profiles offer translational potential in poultry breeding, enabling selection for enhanced traits like bioactive lipids (e.g., linolenic acid) and volatiles (e.g., 1-octen-3-ol) to improve sensory and nutritional quality. In premium markets, these insights enable marketing of differentiated BIAN chicken eggs, appealing for flavor, health benefits, and cultural value, promoting sustainable production and economic opportunities.

## 5. Conclusions

This study conducted a comprehensive analysis to compare the flavor quality and nutritional composition of local BIAN chicken eggs from Shanxi Province with Hy-Line Brown eggs. The analysis integrated GC × GC-TOF MS flavoromics and LC-MS metabolomics. The research revealed significant differences between the two types of eggs. The key finding was that 1-octen-3-ol is a primary compound associated with the distinct aroma of BIAN chicken eggs, exhibiting a much higher Relative Odor Activity Value (ROAV) in BIAN eggs (27.01) compared to Hy-Line Brown eggs (13.46). Additionally, the unique flavor profile of BIAN chicken eggs, characterized by mushroom, green, and sweet notes, is attributed to the synergistic effects of compounds such as heptanal and 2-pentylfuran. Metabolomic analysis revealed that BIAN chicken egg yolks differed markedly in key metabolic pathways such as α-linolenic acid metabolism, cholesterol metabolism, and unsaturated fatty acid biosynthesis, compared to Hy-Line Brown. Specifically, the presence of diverse bioactive lipids (e.g., linolenic acid, 2-linoleoylglycerol) and phenolic compounds (e.g., 3,4-Dihydroxyhydrocinnamic acid, trans-Methylbixin) not only enhances nutritional value but also potentially provides additional health benefits. Enrichment of α-linolenic/linoleic acid metabolism and unsaturated fatty acid biosynthesis increases polyunsaturated substrates for lipid-derived volatiles, while enrichment of cholesterol metabolism and phenylpropanoid biosynthesis respectively reflects active yolk lipoprotein/steroidogenic flux and antioxidant buffering of lipid oxidation, together offering a mechanistic basis for the observed breed-specific flavor and nutritional traits. These results establish a scientific rationale for the preservation and exploitation of indigenous chicken breeds, demonstrating distinct metabolic and flavor profiles that differentiate traditional breeds from commercial varieties. Future studies should investigate genotype-phenotype correlations to identify genetic markers driving these traits and explore epigenetic factors, such as DNA methylation or histone modifications, that may regulate metabolic pathways and flavor compound biosynthesis, informing selective breeding for enhanced egg quality. However, this study is limited by the relatively small sample size (n = 6 per group), single time point analysis (300-day-old hens), and potential confounding factors such as individual metabolic variations and storage effects on volatile compounds, which should be addressed in future research with larger cohorts and multiple sampling points. Subsequent investigations should focus on elucidating the genetic foundations and molecular regulatory mechanisms underlying these exceptional characteristics to offer theoretical insights for enhancing egg quality through precise breeding and nutritional management strategies. Concurrently, efforts to conserve and promote local breeds should be intensified to fully leverage their unique strengths in the contemporary laying hen sector.

## Figures and Tables

**Table 1:** Comparison of ROAV (Relative Odor Activity Value) of flavor compounds in egg yolks of BIAN chicken and Hy-Line Brown chicken.

Name	Class	BC_ROAV	CON_ROAV	Odor Character
2-propyl-Furan	Organo heterocyclic compounds	0.05	0.01	Nuts, sweet
(E)-2-Heptenal	Aldehydes	0.73	0.14	green, fresh, fatty
(E)-2-Octenal	Aldehydes	1.37	0.41	Nuts, Green, Fatty
2-pentyl-Furan	Organo heterocyclic compounds	5.05	1.57	Green Beans, Vegetable
Decanal	Aldehydes	2.45	0.77	tallow, floral, sweet
Pentanal	Aldehydes	7.99	2.81	sickening, rancid, decayed
Heptanal	Aldehydes	5.31	2.53	Citrus, Fatty, Rancid
Butanal	Aldehydes	0.64	0.31	pungent
1-Octen-3-ol	Alcohol	27.01	13.46	Mushroom
2-Octanone	Ketones	0.47	0.28	natural, woody
Styrene	Benzenoids	3.29	0.05	sharp, sweet
1-Butanol	Alcohol	4.76	3.54	sweet, malty, alcohol

Abbreviations: BC, BIAN chicken; CON, Hy-Line Brown chicken; ROAV, Relative Odor Activity Value; CAS, Chemical Abstracts Service.

## Data Availability

The data presented in this study are available on request from the corresponding author.
